# Cognitive activity for the treatment of older adults with mild Alzheimer's Disease (AD) - PACE AD: study protocol for a randomised controlled trial

**DOI:** 10.1186/1745-6215-12-47

**Published:** 2011-02-17

**Authors:** Mandy R Vidovich, Josephine Shaw, Leon Flicker, Osvaldo P Almeida

**Affiliations:** 1 Western Australia Centre for Health and Ageing (WACHA), School of Psychiatry and Clinical Neurosciences & Centre for Medical Research, University of Western Australia and Royal Perth Hospital, Australia

## Abstract

**Background:**

Participation in cognitive stimulation therapy (CST) may reduce the rate of cognitive decline in people with Alzheimer's disease (AD), however it is unclear if the training of carers to deliver activities is sufficient to improve the clinical outcome of patients. The Promoting Healthy Ageing with Cognitive Exercise for Alzheimer's Disease (PACE-AD) study has been designed to determine if change in cognitive function over a six month period can be achieved with participation in cognitive stimulating activities when the intervention is delivered to carers only as opposed to carers and patients.

**Methods/Design:**

The study will aim to recruit 128 community-dwelling men and women with probable AD according to NINCDS-ADRDS criteria. Participants will be randomly allocated to one of two cognitive activity treatment groups: (1) Participants with mild AD and their companions together (2) Companions of participants with mild AD alone. The intervention will consist of a twelve-week program of cognitive stimulation. Seven weeks of the program will involve 90-minute group sessions delivered once per week while the remaining weeks of the program will involve structured home based activities with telephone support. The primary outcome measure of the study is the change from baseline in the total score on the Alzheimer Disease Assessment Scale-Cognitive (ADAS-COG). Secondary outcomes of interest include changes in health related quality of life, mood, memory, language, executive functions, independent living abilities and psychiatric symptoms for participants with mild AD. Changes in companion quality of life, mood, and general health will also be monitored. Primary endpoints will be collected 13 and 26 weeks after the baseline assessment.

**Discussion:**

The proposed project will provide evidence as to whether CST for people with AD and their companions is more beneficial than when used for companions alone. Outcomes sought include a reduction of further cognitive decline and improved quality of life amongst older adults with mild AD. We anticipate that the results of this study will have implications for the development of cost-effective evidence-based best practice to treat people with mild AD.

**Trial registration:**

ACTRN12610000653066

## Background

Cognition-focused interventions are becoming increasingly popular techniques for older adult populations. These interventions are typically grouped into one of three categories - cognitive training, cognitive rehabilitation and cognitive stimulation. Interventions may vary in terms of the degree to which the program is individualised, the content of the activity and the nature of the facilitation (e.g. one-to-one, group, computer based) (see [1] for a review).

Cognitive stimulation therapy (CST) has been recommended as the treatment of choice for individuals in the early stages of dementia (Mini Mental State Examination - MMSE, score ≥ 20) [2]. This type of intervention emphasises the benefits of group activities which, dependent on the target population, can range from education, discussion and debate, and problem solving to reality orientation, reminiscence and validation therapy. In a multi-centred randomised controlled trial (RCT), Spector and colleagues [3] allocated 201 participants with dementia to either a CST group or a no treatment control. People in the intervention demonstrated improved cognitive and quality of life scores. A Cochrane review of reminiscence therapy concluded that whilst there was a need for more rigorous trials, there were promising indications that this form of intervention was of potential benefit to patients and their carers [4]. The cost effectiveness of CST has also been established [5] and manuals operationalised [6].

CST has traditionally been implemented in group settings such as day centres and residential facilities and co-ordinated by trained facilitators. Some interventions have also adopted multi-modal programs targeting cognition and well being in the patient with dementia and addressing the coping abilities and needs of the family member/care-giver with positive results (e.g. [7]). However there is a paucity of research that has utilised cognitive stimulation techniques in a manner easily adopted for the home environment and implemented by the carer/family member.

Quayhagen and Quayhagen [8] had spousal caregivers apply cognitive stimulation techniques and found that improvements in memory, as well as additional aspects of cognition, could be achieved, with a home-based program. This study, however, was limited to spouses and a research team modelled the programme in the home of the dyad. For those patients who are widowed or single, access to a carer or companion may be limited to less contact hours. The feasibility of having instructors/team members provide one-to-one instruction may also restrict the availability of this type of facilitation.

We have designed a single-blind, randomized trial of an intervention drawing on principles of cognitive stimulation. The activities and intervention were selected with regard to suitability for older adults with mild Alzheimer's disease (AD) and designed in a manner that could be readily adapted for use in the home environment and implemented by a companion. The study aims to determine if a program of cognitive activity (CA) that includes both people with AD living in the community and a companion has better cognitive outcomes over six months for participants with dementia than a CA program delivered to companions only. Secondary outcomes for this trial include the quality of life and well being of people with AD and their companions.

## Methods/Design

### Background

The Promoting Healthy Ageing with Cognitive Exercise for the treatment of mild Alzheimer's Disease (PACE-AD) study is a randomised clinical trial that commenced recruitment of participants in October 2009.

In 2007, our group developed a cognitive activity intervention program which was well received by older adults with mild cognitive impairment [9]. In early 2009, this program was modified for its suitability for participants with mild AD. Nine relatives of individuals with mild AD were invited to attend a morning education and activity session regarding the proposed study. Feedback from the relatives regarding the nature of the study was overwhelmingly positive, though the length of the proposed sessions, coupled with the complexity of the program content, were areas identified as requiring further consideration. After collating the verbal and written feedback, additional minor modifications were made to the study protocol and recruitment began.

### Study Design and Setting

PACE-AD is a six-month, single blind randomised trial of a CA intervention delivered to older adults with mild AD and companions compared with companions alone.

### Ethics

The Human Research Ethics Committees of Royal Perth Hospital (RPH), Mercy Hospital and Bentley Hospital have approved the study protocol and procedures, and the study is being conducted according to the Declaration of Helsinki.

### Recruitment and Selection of participants

Recruitment for this trial is currently ongoing. Participants are community dwelling volunteers, recruited mainly from local memory clinics. Potentially suitable participants are approached via mail and receive a follow up telephone call. Interested volunteers are screened with a semi-structured telephone interview and invited to visit the Mercy Hospital for a more detailed screening assessment (clinic screen) and to provide written informed consent.

#### Inclusion and Exclusion Criteria

The defining feature of participants included in the PACE-AD study is a diagnosis of Alzheimer's disease (probable or possible) according to the National Institute of Neurological and Communicative Disorders and Stroke and the Alzheimer's Disease and Related Disorders Association (NINCDS-ADRDA) Alzheimer's Criteria. Participants need to have a Mini Mental State Examination (MMSE) score between 18-26 inclusive (i.e., mild severity), at the time of screening and be fluent in written and spoken English.

Mild AD participants with a prevalent psychiatric disorder (e.g. depressive episode), current history of hazardous or harmful alcohol consumption (Alcohol Use Disorders Identification Test - AUDIT - score ≥15 [10]), or who do not have an available companion are excluded. Those individuals with a current medical condition preventing participation in the study tasks (such as severe sensory impairment) or associated with reduced survival over a six-month period (e.g. advanced cancer) are also excluded.

#### Telephone Interview

Volunteers are initially screened via a telephone interview to ascertain suitability for the study. Those without a suitable companion (someone who spends at least ten hours per week with the person, including time at their home) are immediately excluded. All individuals are asked about their general health (past and current), education, English literacy skills and current alcohol and cigarette consumption. Telephone interviews range from 10 to 20 minutes in length and those meeting provisional inclusion criteria are invited to a face-to-face assessment (clinic screen).

#### Clinic Screen

After obtaining written consent from volunteers with mild AD and their companions each person's eligibility for the study is established according to the following criteria:

##### Mild AD

• Mini Mental State Examination score ranging between 18-26 inclusive [11]

• Patient Health Questionnaire (PHQ-9) score <15 [12]

##### For the companion of the volunteer with mild AD

• MMSE total score of 26 or above [11]

• PHQ-9 score <15 [12]

The MMSE[11] is a brief test of mental status and cognitive function commonly used to screen for dementia and to monitor cognitive decline. It produces a total score that can range from 0 to 30. Scores lower than 24 are reliably associated with the diagnosis of dementia or other organic mental disorders. The present study also used the MMSE to exclude companions with cognitive impairment. The PHQ-9[12] is a widely used scale to establish the presence of clinically significant depression amongst community-dwelling adults. Scores range from 0 to 27, with scores of 15 or greater indicative of clinically significant depression.

In addition to the cognitive and mood screen, a self-reported medical history questionnaire is completed by each individual, which includes details regarding their medication usage. This information is also collected at the final assessment. The screening assessment of both volunteers takes approximately 30 minutes to complete and any pertinent clinical information is reported to the relevant treating physician with the consent of the study participant. If both volunteers meet criteria for the study, they proceed to undertake the baseline assessment.

### Outcome Measures and Assessment Procedures

#### Baseline and Follow-up Assessments

**Figure 1:**
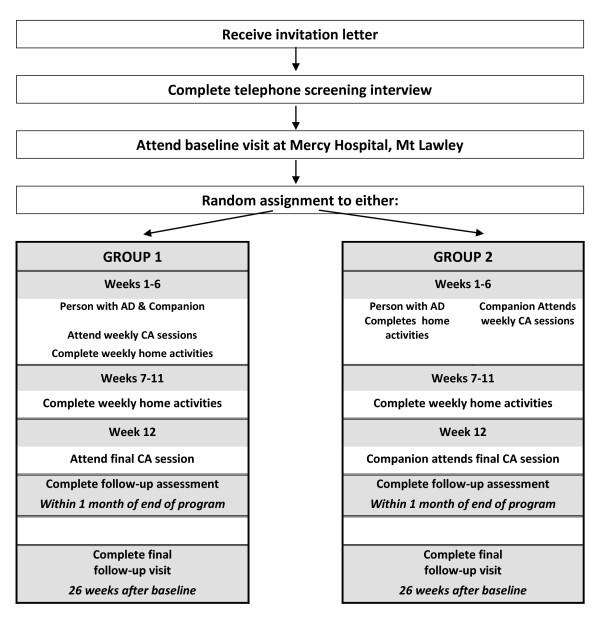
Baseline assessment

Baseline assessments are completed immediately after the clinic screen, 1-2 weeks prior to the first intervention session and randomisation. Post-intervention assessments are undertaken within two weeks of program completion and the final assessment is completed 26 weeks after the baseline assessment (see Figure 1). All assessments take between 90 to 120 minutes to complete (including the provision of short breaks) and consist of the following series of tests and questionnaires (see also Table 1).

**Table 1:** Outline of the primary and secondary outcome measures used in the PACE-AD trial

*Assessment Tool*	Participant with mild AD	Companion
ADAS-COG	X	

RBMT-3	X	

ToL	X	

COWAT	X	

MMSE	X	X

PHQ-9	X	X

DEMQOL-version 4	X	X

Short-Form IQ CODE	X	X

IADL		X

NPI-Q		X

AUDIT		X

SF-12		X

The X indicates who completed the respective assessments (participant with mild AD or companion). All measures were given at baseline, 13 and 26 weeks.

ADAS-COG = Alzheimer Disease Assessment Scale-Cognitive; RBMT-3 = Rivermead Behavioural Memory Test-Third Edition; ToL = Tower of London; COWAT = Controlled Oral Word Association Test; MMSE = Mini Mental State Examination; PHQ-9 = Patient Health Questionnaire - Nine Item; Short-Form IQ CODE = Modified Short Form of the Informant Questionnaire on Cognitive Decline in the Elderly; IADL = The Lawton Instrumental Activities of Daily Living; NPI-Q = The Neuropsychiatric Inventory Questionnaire; AUDIT = World Health Organisation's Alcohol Use Disorders Identification Test; SF-12 = Short-Form 12-Item Health Survey.

#### Primary outcome measure

Alzheimer Disease Assessment Scale-Cognitive (ADAS-COG)[13] is a frequently used measure of global cognitive functioning and assesses cognitive domains including orientation, memory, language, and praxis; common areas of impairment present in AD. It provides sub-scale scores as well as a global score out of 70, with higher scores indicating lower levels of cognitive functioning. A four-point change on the ADAS-COG over a six month period is considered a clinically relevant difference [14,15].

#### Secondary Outcome Measures

##### Measures completed by participants with mild AD

The Rivermead Behavioural Memory Test-Third Edition (RBMT-3)[16] comprises a series of memory tasks analogous to those faced in everyday situations. Sensitive to changes in memory functioning over time and well validated with patient groups [16], a parallel version also reduces the confounds of practice effects. Three subtests from the RBMT-3 are performed with mild AD participants at all assessment time points, with the parallel version administered at the post intervention assessment.

The Tower of London (ToL) [17] consists of ten problems of increasing difficulty designed to assess executive planning abilities. The participant is required to manipulate three coloured beads across three pegs on a wooden board to mirror the examiner's board. Participants are instructed to solve the problem in as few moves as possible while adhering to two specific rules, within a two minute time limit. In the current trial this test is administered with minor modifications, in order to minimise frustration for participants with mild AD.

The Controlled Word Association Test (COWAT) [18] requires the participant to generate as many words as possible with a given letter of the alphabet, within a one-minute time period, excluding proper nouns and the same word with a different suffix. This test is used as an indicator of executive functioning for participants with mild AD.

##### Measures completed by participants with mild AD and their companions

We use the MMSE total score and the PHQ-9 total score, as previously described, to monitor changes in cognition and mood of all participants throughout the trial.

DEMQOL-version 4[19] is a 28-item self-reported, interviewer administered questionnaire assessing participants' perceptions of their health related quality of life in the past week. It assesses five domains: daily activities/self care, health and well-being, cognitive functioning, social relationships, and self-concept. The DEMQOL has been demonstrated to show high reliability (internal consistency and test-retest) and moderate validity in people with mild to moderate dementia. In this trial, participants with mild AD are given the questions verbally with the aid of visual response options. Companions' quality of life is also assessed with this measure.

Modified Short Form of the Informant Questionnaire on Cognitive Decline in the Elderly (Short-Form IQ Code) [20] is an informant based, brief cognitive screening questionnaire for individuals with dementia. It assesses an individual's cognitive abilities as they apply to everyday situations and consists of 16 items. The original version asks the informant to judge the extent to which the patient's level of functioning has changed in the past ten years. Subsequent versions have used different time frames to allow for informants who might not have known the patient for very long. In this trial only AD participants' current level of functioning will be assessed, to allow direct comparison at follow-up testing.

##### Measures completed by companions

The Lawton Instrumental Activities of Daily Living (IADL) [21] scale is an informant based questionnaire which assesses the patient's current ability to perform eight independent activities of daily living. These include using the telephone, shopping, food preparation, housekeeping, laundry, mode of transportation, taking medications and handling finances.

The Neuropsychiatric Inventory Questionnaire (NPI-Q)[22] is a brief screening instrument assessing the frequency and severity of twelve neuropsychiatric behavioural disturbances common in dementia. The companion is asked to rate the presence, change and severity of symptoms in the AD patient in the past month along with associated caregiver distress. The NPI-Q is considered to be a reliable and valid tool for assessing psychopathology in dementia patients and is sensitive to treatment effects.

The World Health Organisation's Alcohol Use Disorders Identification Test (AUDIT) [10] is used to screen for risky, hazardous or harmful drinking. There are 10 items and supplementary questions, with questions scored on a scale of 0 to 4. Scores of 16 or above suggest "high-risk" or "harmful level" of drinking behaviour. This questionnaire is monitoring the companion's alcohol usage throughout the trial.

Short-Form 12-Item Health Survey (SF-12) [23] is a self-report questionnaire consisting of twelve questions from the SF-36 Health Survey [24] and assesses an individual's perception of their general physical/health functioning, bodily pain, vitality, social functioning, general mental health, psychological wellbeing, and role limitations due to physical or emotional issues. This questionnaire is used to monitor change in the companion's mental and physical components of quality of life throughout the trial.

#### DNA Collection

Participants with mild AD are asked to provide a saliva sample for the extraction of DNA to assess the influence of common genetic polymorphisms (e.g., apolipoprotein E4 genotype) on the outcomes of the study. The samples are collected and processed by the Department of Clinical Pathology and Biochemistry at the RPH and stored at -80°C. All material is batched and will only be processed at the end of the trial.

### Intervention

Following the baseline assessment, dyads (person with mild AD and companion) are randomised to one of two intervention groups. The intervention consists of a twelve-week CA program. Sessions are run by facilitators experienced in conducting research with older adults. The two groups are exposed to the same length of intervention, social interaction and contact with the program facilitators. The program was developed by a qualified Neuropsychologist (MV) and a manual produced. All of the sessions are delivered in a structured way for consistency, and audio-taped for subsequent fidelity assessment.

Research assistants (RAs) blinded to group allocation conduct all assessments. RAs are provided with strict instructions to avoid any potential opportunity for disclosure regarding intervention participation. Following completion of the trial, RAs undertaking data collection will be asked to identify the group membership of participants, to determine the effectiveness of the blinding procedures that were put in place for this project. A brief summary of each intervention group is provided below.

Participants with mild AD and their companions (Group 1): Each group consists of a maximum of five dyads taking part in 90-minute sessions once a week for seven weeks. Session One introduces the nature of the program and develops familiarity within the group, with personal introductions and sharing of background information and experiences. Sessions Two to Six focus on defining attention, processing speed, memory, language and executive functions. These sessions outline how these cognitive abilities are affected in AD and provide participants with strategies and techniques for managing declining capacity in each of these domains. Regular opportunity for supervised practice of such techniques and examples of activities to strengthen abilities occurs in all sessions. Dyads are also provided with an hour of home activities, to reinforce material learnt in the sessions. Sessions Seven to Eleven are completed by the participant with mild AD and their companion together in their own environment. Participants are provided with a workbook containing instructions and examples, along with phone calls from the facilitator once per week to address any questions and to monitor task completion. The final session (Session 12) offers an overview and discusses strategies to maximise participation.

Companions of participants with mild AD alone (Group 2): This group consists of a maximum of five companions presented with the same session information and materials as Group 1. The only difference is that the companions attend the sessions with other companions, and are instructed to convey what is learnt during the sessions to the participant with mild AD during their home activities.

### Randomisation

Randomisation is performed according to a random list of numbers generated by computer and undertaken in random blocks of 8 or 10 with no more than five dyads allocated to each group. The allocation list is handled by an independent investigator (OPA) who has no contact with study participants and is not involved in the supervision of staff responsible for the collection of data. The allocation table is then passed on to the facilitator running the intervention, who invites eligible participants to join the relevant groups. RAs undertaking the follow-up assessments remain blinded to group allocation.

### Sample Size and Power Calculation

This trial aims to recruit 128 participants with mild AD and their carers, with 64 patients being allocated to each study group. A study of this size will have 80% power to detect between group differences on the ADAS-COG associated with moderate effect size (Cohen's d = 0.5) and alpha of 5%.

### Analysis of the Data

Changes in the ADAS-COG score from baseline are the primary outcome of interest in the study. We will model these changes at 2 time points: 13 (immediately after the intervention comes to an end) and 26 weeks. We will use mixed effects models to analyse the data. This approach will enable us to take into account the cognitive performance of participants at baseline, as well as the intra-person correlation generated from repeated measures. Intention-to-treat (ITT) analyses will be based on the use of imputation by chain equations (ICE), which will precede the use of the mixed-effects model. The ITT will be the primary analysis of the study.

## Discussion

Cognition-focussed interventions are increasingly being adopted for the treatment of cognitive decline in older adults, with CST recently recommended as the treatment of choice for individuals with mild dementia [2]. However, the efficacy, sustainability of effect, and cost-effectiveness of involving and training a carer/companion to deliver cognitive tasks, and the degree to which cognitive changes can be maintained over time using this form of delivery, is yet to be established.

This trial has been designed according to CONSORT guidelines and has been structured to enable its reproduction in both research and clinical settings. We expect to complete recruitment by December 2011 and anticipate that the results of this study will have implications for health care policy and resourcing and facilitate improvements in the management of people with mild AD. The results of this study will enable us to determine, for the first time, if a CA intervention delivered to companions alone is as effective at promoting changes in cognitive function as an intervention involving both the person with dementia and his/her companion. These results will have important implications for the design of sustainable cost-effective health services for people with mild AD.

## Competing interests

The authors declare that they have no competing interests.

## Authors' contributions

All authors are members of the PACE-AD project group. MV acts as guarantor of the data; she has designed the intervention and supervises research staff. MV and OPA designed the study, which is funded by a grant from ANZ Trustees Limited to OPA. JS is responsible for data collection and contributed to drafting the paper together with MV. OPA and LF reviewed the manuscript critically. All authors have approved the submission of the present paper to Trials.
